# Reduced anterior cingulate grey matter volume in painful hand osteoarthritis

**DOI:** 10.1007/s00296-018-4085-2

**Published:** 2018-06-23

**Authors:** Mark D. Russell, Thomas R. Barrick, Franklyn A. Howe, Nidhi Sofat

**Affiliations:** 1 Institute for Infection and Immunity, St George’s, University of London, London, SW17 ORE UK; 2 Molecular and Clinical Sciences Research Institute, St George’s, University of London, London, SW17 ORE UK

**Keywords:** Hand osteoarthritis, Pain sensitisation, Pregabalin, Duloxetine

## Abstract

**Objective:**

Increasing evidence supports the role of central sensitisation in osteoarthritis (OA) pain. In this study, we used neuroimaging to compare pain-processing regions of the brain in participants with and without hand OA. We then assessed for volumetric changes in these brain regions following treatment with centrally acting analgesics.

**Methods:**

Participants with hand OA (*n* = 28) underwent T1-weighted MRI of the brain before and after 12 weeks of treatment with pregabalin, duloxetine or placebo. Grey matter volume in the anterior cingulate cortex (ACC), insular cortex and thalamus was compared to non-OA control subjects (*n* = 11) using FreeSurfer regional volumetric analysis and voxel-based morphometry, and evaluated for differences pre- and post-treatment.

**Results:**

Relative to non-OA controls, hand OA participants had areas of reduced grey matter volume in the ACC at baseline (*p* = 0.007). Regional volumetric differences in the ACC persisted after 13 weeks’ treatment with pregabalin or duloxetine (*p* = 0.004) with no significant differences between treatment cohorts, despite improvements in NRS pain scores for pregabalin (*p* = 0.005) and duloxetine (*p* = 0.050). The ACC grey matter changes persisted despite a significant improvement in pain in the pregabalin and duloxetine groups vs. placebo. No structural differences were observed in the insular cortex or thalamus at baseline or following treatment.

**Conclusion:**

Our study found evidence of reduced ACC grey matter volume in participants with hand arthritis that persisted after treatment with centrally acting analgesics pregabalin and duloxetine, respectively. The sustained changes observed in the ACC in our study could reflect the relatively short duration of treatment, or that the differences observed are irreversible volume changes due to chronic pain that are established over time.

**Electronic supplementary material:**

The online version of this article (10.1007/s00296-018-4085-2) contains supplementary material, which is available to authorized users.

## Introduction

Osteoarthritis (OA) of the hand is a major cause of pain and functional impairment worldwide, with many people experiencing chronic pain despite treatment with paracetamol, non-steroidal anti-inflammatory drugs (NSAIDs) and opioids. An increasing number of studies implicate abnormalities in brain regions associated with pain processing in the maintenance of chronic pain in OA and other conditions [1–3]. Using functional MRI, it has been shown that pain-processing areas of the brain, including the cingulate cortices, insular cortices and thalamus, are activated in hand OA patients but not control subjects during a finger-flexion task [4]. Grey matter volumetric changes have also been demonstrated in pain-processing brain regions in patients with hip and knee OA and other chronic pain conditions, with the pattern of brain involvement varying according to the underlying condition [1, 5–9]. Amelioration of hip OA pain through arthroplasty increased thalamic grey matter volume in one study [5], and increased anterior cingulate cortex (ACC) and insular cortex grey matter volume in another study [6]. These findings suggest that there can be neuroplasticity, rather than purely neurodegenerative effects, involved in chronic pain. They also provide a rationale for the investigation of centrally acting analgesic agents as treatment options for chronic OA pain; supported by studies demonstrating pain improvements following treatment with pregabalin and duloxetine [10–12].

We hypothesised that structural differences in the ACC, insular cortex and thalamus would be present between hand OA participants and non-OA control participants, and that treatment with centrally acting analgesics would lead to volumetric changes in these brain regions. To investigate this hypothesis, we obtained volumetric brain MRI from hand OA participants before and after treatment with pregabalin, duloxetine or placebo, and compared regional brain volumes with non-OA control participants.

## Methods

This brain neuroimaging work was part of a randomised, double-blinded, placebo-controlled study, designed to assess the effects of pregabalin and duloxetine on pain outcomes. The clinical endpoints of this study, in addition to the full trial protocols, have been reported elsewhere [10, 11]. All trial protocols were approved by the study sponsor and the MHRA (Medicines and Healthcare products Regulatory Agency), UK. All procedures performed in studies involving human participants were in accordance with the ethical standards of the institutional and/or national research committee and with the 1964 Helsinki Declaration and its later amendments or comparable ethical standards. Ethical approval for this study was provided by the London-Surrey Borders Ethics Committee, with approval number 12/LO/0047. Informed consent was obtained from all individual participants included in this study.

Clinical Trial Registration Number: NCT02612233.

The trial protocol was followed as published, according to CONSORT guidelines and inclusion–exclusion criteria.

### Participants

Study participants were recruited from primary care and rheumatology outpatient clinics in London, UK. Participants were eligible for inclusion if they were aged 40–75 years, had hand OA diagnosed by American College of Rheumatology criteria, with pain of at least five or above on a Numerical Rating Scale (NRS) of 0–10. Participants were receiving usual standard of care for hand OA, including paracetamol and/or NSAIDs. Exclusion criteria were the presence of other rheumatological diagnoses; contraindications to, or previous use of, duloxetine or pregabalin; concurrent use of opioids; current or planned pregnancy; uncontrolled depression; recent surgery; ischaemic heart disease; diabetes mellitus; excessive alcohol intake; chronic kidney disease; hepatic impairment and hypertension. Volunteers with no evidence of symptoms of hand OA were recruited, 11 of whom were selected as neuroimaging control subjects for this study on the basis of having similar mean ages and age ranges to the hand OA participants. The control subjects did not report any pain, anxiety or depression.

### Study groups

Participants with hand OA were randomised to receive one of three study treatments: pregabalin, duloxetine or placebo. Before the treatment, all groups were homogeneous for pain reporting measures, concomitant medication use and duration of diagnosis, as previously published [10]. All three treatments were administered using a dose escalation/de-escalation protocol over a period of 12 weeks: week 1: pregabalin, 150 mg once nightly (ON), vs. duloxetine, 30 mg ON, vs. placebo, 1 capsule ON; weeks 2–11: pregabalin, 300 mg ON, vs. duloxetine, 60 mg ON, vs. placebo, 2 capsules ON; week 12: pregabalin, 150 mg ON, vs. duloxetine, 30 mg ON, vs. placebo, 1 capsule ON; followed by cessation of therapy. Neuroimaging control subjects were not administered any treatment. Study drugs were supplied by Sharp Clinical Services (formerly Bilcare GCS (Europe), Powys, UK), which over-encapsulated pregabalin 150 mg tablets or duloxetine 30 mg tablets and produced visually identical placebo capsules. The random allocation sequence, with a block size of nine, was generated by the manufacturer and implemented through sequentially numbered containers. Neither participants nor investigators were aware of treatment assignment until after completion of the trial, which was performed after the last patient and last visit was conducted at the end of the trial. Ongoing use of paracetamol and/or NSAIDs was permitted during the study period. Our sample size was based on the IMMPACT guidelines [11] for randomised controlled trials using the validated 0–10 point pain numerical rating scale (NRS) for the sample size calculation and primary outcome measures, and is reported in our published paper [10].

Participants enrolled into the main clinical study described above [10] were subsequently assessed for MRI eligibility using a standard clinical safety checklist. Participants were ineligible for MRI if they had a cardiac pacemaker, pacing wires, artificial heart valve, previous brain haemorrhage with clipping or coil insertion, hydrocephalus shunt, history of work with high-speed metal machining tools, recent joint replacement or other contraindicated implants, e.g. cochlear implants. Repeat neuroimaging was performed on hand OA participants at 13 weeks, after completion of study treatment. Neuroimaging control subjects did not receive study treatment and were not re-imaged at 13 weeks.

### Procedures

The primary endpoints were the numerical rating scale (NRS) and the Australian and Canadian Hand Osteoarthritis Index (AUSCAN) rating scale for pain after 13 weeks’ treatment, which has been reported in the clinical trial outcome data elsewhere [10]. NRS and AUSCAN pain measures, as well as HADS anxiety and depression scores, were recorded at baseline and after 13 weeks’ intervention for all OA study participants.

High-resolution 3D T1-weighted images were acquired at baseline from all eligible hand OA and non-OA participants and also after treatment at the 13-week time point for participants with OA. Images were obtained with a 3T Philips Dual Tx Achieva using a 32-channel head coil and a 3D Turbo Field Echo with parameters: TE = 4 ms; TR = 8 ms; flip angle = 8°; a pre-inversion pulse; echo train length = 240; a 240 mm field-of-view in the sagittal orientation with 1 mm in-plane resolution and a 160 mm field of view in the left–right direction with 107 slices of 1.5 mm thickness. A sensitivity encoding (SENSE) factor of 2.0 and 0.75 k-space sampling factor resulted in a 3.8 min total acquisition time.

### Regional volumetric analysis

Tissue volumes of a priori regions of interest (ROI) were obtained using FreeSurfer version 5.3 [13] and statistical comparisons made with SPSS version 22 (IBM Corp). The ACC (with separate caudal and rostral regions in FreeSurfer), insular cortex and thalamus of each hemisphere were delineated for each participant using the inbuilt DKTAtlas40 cortical and Aseg subcortical atlases.

### Voxel-based morphometry

Voxel-based morphometry (VBM) was performed using SPM12 to detect significant localised changes in tissue volume within the a priori ROIs [14]. Pre-processing steps included segmentation into grey matter, white matter and cerebrospinal fluid using SPM. The native grey matter images were skull stripped and spatially normalised to Montreal Neurological Institute (MNI) space using a study-specific template. These images were Jacobian-scaled and smoothed with an isotropic Gaussian kernel of 6 mm full width at half maximum. Image masks were created for the a priori ROIs (ACC, insular cortex and thalamus of each hemisphere) using the WFU PickAtlas toolbox (http://fmri.wfubmc.edu/*)*.

### Statistical analysis

The IBM SPSS Statistics 25 package was used for all statistical analysis apart from that which was implicit to the volumetric analyses of the MRI data as described above. Analysis of clinical and MRI parameters was performed on the patient subgroup from the main clinical trial [10] for which there were both pre and post-treatment MRI scans, and in comparison to an age-matched group of healthy controls who had an MRI scan with no subsequent treatment. A one-way ANOVA was used to assess whether there were demographic differences between patient and control groups. An ANOVA using a general linear model with repeated measures across all treatment groups was used to assess whether there were treatment related effects of clinical scores or brain volumes. A post hoc analysis using paired* t* tests was used to assess whether there were significant differences in clinical scores and brain volumes for each treatment group. Two-sample* t* tests were used to assess whether there were significant differences in brain volumes between OA patients and controls. For the comparison of control and OA patient brain volumes, age and total intracranial volume were used as necessary covariates.

#### Regional volumetric statistical analysis

A mixed linear effects model was used to compare volumes between patient and control groups, with age and total intracranial volume as covariates. Repeated measures ANOVA were used to assess treatment-associated volumetric differences in the a priori ROIs between the pregabalin, duloxetine and placebo cohorts. Results were considered statistically significant if the two-tailed* p* values obtained were less than 0.05.

#### Voxel-based morphometry statistical analysis

Structural comparisons between patients and controls were performed with a two-sample* t* test, covarying for age and total intracranial volume. Paired* t* tests were used for comparisons of patients before and after treatment. All voxel-based statistics were performed in SPM12. Corrections for multiple comparisons within the ROIs were performed using family-wise error (FWE) at peak level and cluster level. Findings are displayed as uncorrected and FWE-corrected, two-tailed *p* values, with thresholds of statistical significance set at *p* < 0.001 and *p* < 0.05, respectively.

## Results

### Study participants and demographics

Thirty-nine participants were recruited from the total of 65 participants enrolled into the main clinical trial who were eligible for and agreed to undergo MRI scanning. The full clinical trial results are published [10, 11]. Of the 39 patients who underwent a baseline MRI, 30 patients agreed to have a post-treatment MRI scan. The MRI data from two hand OA participants were further excluded due to image artefacts, leaving 28 hand OA participants with MRI data available for pre- and post-treatment analysis and 11 controls in an aged matched group for comparative analysis. Randomisation of hand OA participants into treatment cohorts occurred as part of the main clinical study, prior to assessment of eligibility for inclusion into this MRI sub-study. Differences were, therefore, present in the numbers of hand OA participants with MRI data in each of the three treatment cohorts: duloxetine (*n* = 11), pregabalin (*n* = 6), placebo (*n* = 11). The demographics of the hand OA and control groups are shown in Table 1 and there were no significant differences between control and patient groups. In addition, ANOVA across the three treatment groups of patients showed no significant differences for age, baseline NRS and AUSCAN pain scores or HADS anxiety and depression scores (*p* > 0.2 for all measures).

**Table 1:** Demographics of hand OA participants and control participants used in the MRI analysis

Study demographic	Hand OA participants	Control participants	*p* value for differences between groups
Number of subjects	28	11	
Age (years), mean (SD)	62 (7.7)	59 (7.4)	0.18
Women (%)	24 (86)	9 (82)	0.77
Handedness	27 Right 1 Left	11 Right 0 Left	0.54

### Regional volumetric analysis at baseline

**Fig. 1:**
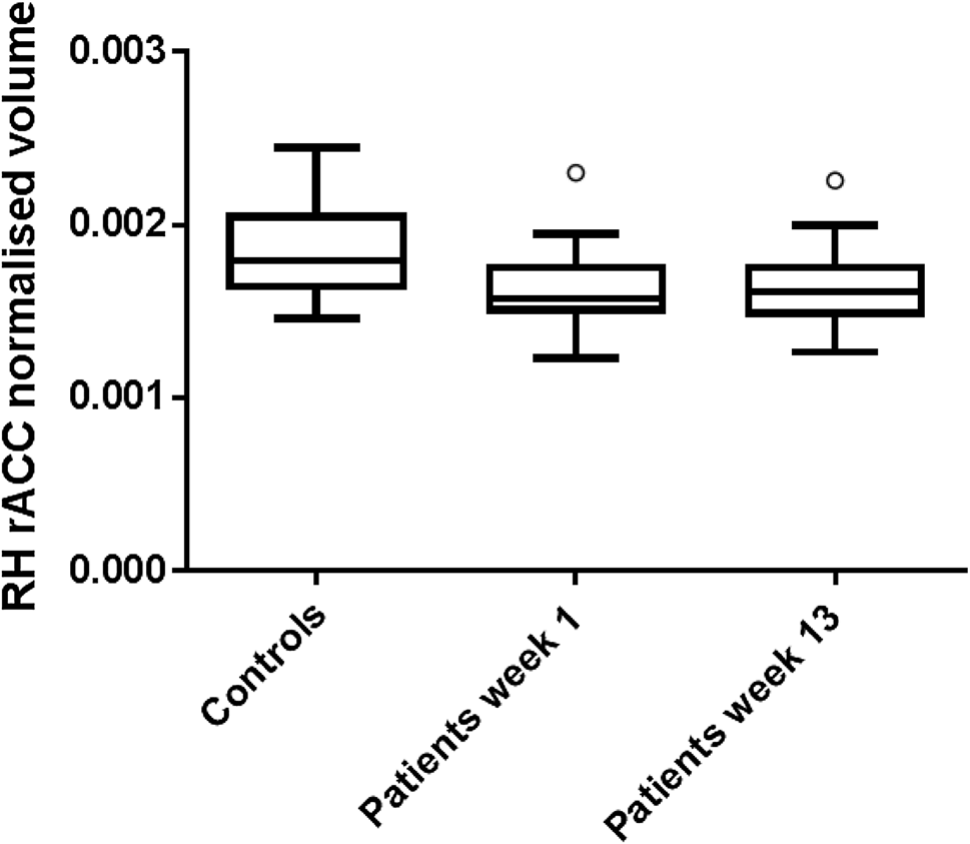
Boxplot representation of grey matter volume in the right hemispheric rostral anterior cingulate cortex (RH rACC) of control subjects (*n* = 11) and subjects with hand OA (*n* = 28) undergoing MRI at baseline and after completion of study treatment (13 weeks). Relative to controls, RH rACC grey matter volume was lower in hand OA subjects at baseline (*p* = 0.007) and at 13 weeks (*p* = 0.004) using a mixed effects linear regression model with age and total intracranial volume as covariates. Volumes were derived from FreeSurfer regional volumetric analysis and have been displayed graphically as normalised to total intracranial volume. Median volumes are displayed in the boxplot, along with upper and lower quartiles. Inner fences represent 1.5 times the interquartile range and circles represent outlier data

Using FreeSurfer regional volumetric analysis [13], we assessed for baseline differences between hand OA and control participants in the a priori ROIs (the ACC, insular cortices and thalami). Grey matter volume in the right hemispheric rostral ACC was 14% lower in hand OA participants than control participants at baseline (*p* = 0.007) (Fig. 1). No significant baseline volumetric differences were evident in the insular cortices or thalami.

### Voxel-based morphometry at baseline

**Fig. 2:**
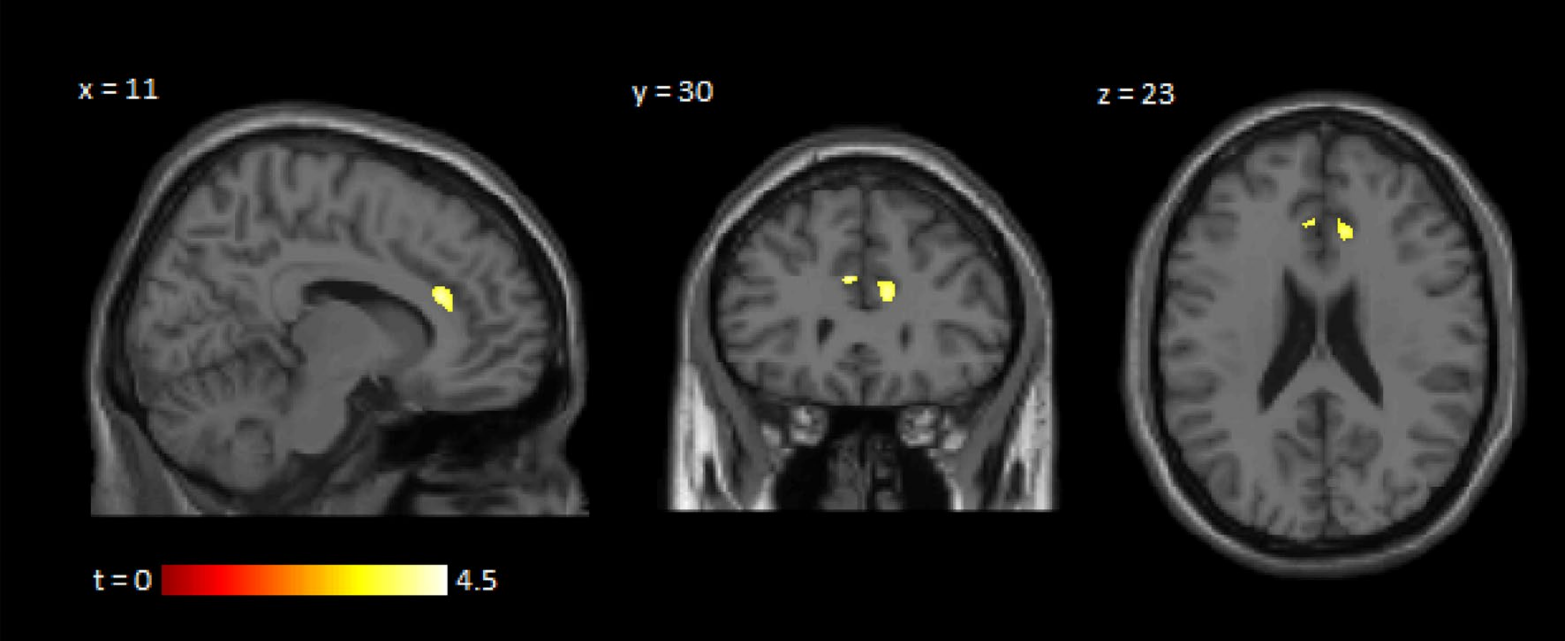
Voxel-based morphometric analysis demonstrating clusters of reduced grey matter volume in the ACC of participants with hand OA, relative to control subjects, at baseline. The right side of the figure represents the right side of the brain. Clusters are indicated on a brain template with corresponding slice coordinates and a *t* test significance scale. The right hemispheric ACC cluster corresponds to 270 voxels using an uncorrected significance threshold of *p* < 0.001 (*p* = 0.072 when FWE-corrected for multiple comparisons at peak level; *p* = 0.011 when FWE-corrected for multiple comparisons at cluster level; MNI coordinates: *x* = 11, *y* = 29, *z* = 20). The left hemispheric ACC cluster corresponds to 65 voxels using an uncorrected significance threshold of *p* < 0.001 (*p* = 0.020 when FWE-corrected for multiple comparisons at peak level; *p* = 0.114 when FWE-corrected for multiple comparisons at cluster level; MNI coordinates: *x* = − 4, *y* = 31, *z* = 25)

Voxel-wise comparisons were performed using VBM [14] to identify sub-clusters of reduced grey matter within the ACC of hand OA subjects at baseline, relative to control subjects. In the right hemispheric ACC, a cluster of 270 voxels was evident with an uncorrected significance threshold of *p* < 0.001. In the left hemispheric ACC, a cluster of 65 voxels was evident with an uncorrected significance threshold of *p* < 0.001 (Fig. 2; see legend for FWE-corrected *p* values and MNI coordinates). No significant volumetric differences were evident in the insular cortices or thalami.

### Post-treatment analyses

The effects of treatment on MRI derived brain volumes and clinical scores of pain were assessed with ANOVA using a general linear model with repeated measures across all treatment groups. Repeated measures ANOVA revealed no significant regional volumetric changes between treatment cohorts in the ACC, insular cortex or thalamus. Similarly, VBM analyses showed no significant treatment-associated volumetric changes in the a priori ROIs. Relative to non-OA controls, the volumetric differences in the right hemispheric rostral ACC of hand OA participants were still evident on repeat imaging at 13 weeks, after completion of study treatment (*p* = 0.004) (Fig. 1).

Repeated measures ANOVA of the pain scores indicated *p* = 0.068 for the treatment response measured using NRS and *p* = 0.021 of that measured by AUSCAN pain. There were no significant differences in response between treatments, but the pregabalin-treated cohort showed the greatest reduction in pain score (see supplementary Figs. 1 and 2) as also observed in the main clinical study [10].

Paired *t* tests were used post hoc to assess differences in clinical pain (see below) and in anxiety and depression scores (Supplementary Table). Baseline NRS for pain was 6.64 in the duloxetine cohort, 5.83 in the pregabalin cohort and 6.45 in the placebo cohort. After 12 weeks of treatment, a significant reduction in pain was seen in the pregabalin cohort (mean NRS pain reduction of 2.83; 95% confidence interval 1.29–4.38; *p* = 0.005). With duloxetine, a reduction in pain of borderline statistical significance was noted (mean NRS pain reduction of 1.45; 95% confidence interval 0.00–2.91; *p* = 0.050). No significant change in pain was seen with placebo (mean NRS pain reduction of 0.64; 95% confidence interval − 0.33–1.60; *p* = 0.171).

Baseline AUSCAN pain was 292 in the duloxetine cohort, 311 in the pregabalin cohort and 315 in the placebo cohort. After 12 weeks of treatment, a significant reduction in pain was seen in the pregabalin cohort (mean AUSCAN pain reduction of 132; 95% confidence interval 48–217; *p* = 0.01). There were no significant changes in AUSCAN pain for duloxetine or placebo (see Supplementary Table).

Hospital Anxiety and Depression Scale (HADS) anxiety and depression scores were not significantly different following treatment with pregabalin or duloxetine (see supplementary table). Hand OA participants receiving placebo had no change in their HADS depression scores but higher HADS anxiety scores following treatment (*p* = 0.031), albeit still below the threshold score for mild anxiety.

## Discussion

The data we report in this study are a brain MRI subgroup analysis of a clinical trial which compared centrally acting analgesics pregabalin and duloxetine to placebo in hand OA. In this sub-study, we found the same pattern of treatment response as in the main clinical study, with a significant reduction in clinical pain scores for those patients treated with pregabalin. Specific to this MRI study, we found at baseline that participants with hand OA demonstrated reduced grey matter volume in the ACC relative to non-OA control participants. Our study is the first, to our knowledge, which reports structural brain changes in hand OA and supports the hypothesis of central sensitisation in hand OA. The evidence of structural changes in hand OA in brain regions associated with pain processing supports the notion that central pain processing is a therapeutic target in chronic painful conditions such as hand OA. However, this volume difference persisted despite a treatment induced reduction in clinical pain score, suggesting that the action of the treatment may be affecting brain regions other than those that appear reduced in OA patients.

This contrasts findings from studies of hip OA, in which at least partial reversal of brain volumetric changes was observed following hip arthroplasty [5–7]. In one study, thalamic volume increases were noted 9 months after hip arthroplasty [5]. In another study, postsurgical increases in ACC and insula volume became evident beyond 12 weeks [6]. The differences in findings between our study and the surgical studies may reflect the different pain relief methods employed, or the better analgesic outcomes in the surgical studies. They could also reflect the shorter duration of follow-up in our study, which might have precluded detection of delayed grey matter changes. Alternatively, the ACC changes in our cohort of hand OA participants may represent an irreversible maladaptive neuroplasticity of a chronic pain state. Longitudinal studies with longer follow-up periods after treatment with agents such as pregabalin and duloxetine are required to further investigate this. Recent work by other groups has suggested a significant negative association between the ‘pain score’ component and regional cerebral blood flow to a right temporal lobe cluster, including the amygdala and the parahippocampal cortex in a study of hand OA [15]. Tetrault et al. [16] found volumetric (and fMRI) changes that are associated with a reduction in pain for knee OA treated with placebo and duloxetine do not overlap within regions of grey matter that are different between participants with OA and non-OA controls. In their recent study in 39 participants with knee osteoarthritis (OA) patients (22 females), randomised to duloxetine (60 mg once daily) or placebo, Tetrault et al. [16] showed that outcomes for pain relief were equivalent between treatment groups. The group suggested that distinct circuitry changes in the brain could explain pain relief in each group in brain measures that included grey matter density and resting state fMRI nodal degree count.

It has been proposed that the ACC integrates multiple facets of the pain response, including the affective, anticipatory and cognitive components [2, 17]. Morphometric studies from several chronic pain conditions have identified the ACC as a focus of structural change, with reductions in grey matter volume frequently seen [1, 2, 6, 7]. In functional neuroimaging studies, ACC activation is seen in response to painful stimuli in both healthy subjects and subjects with chronic pain conditions [17]. Deep brain stimulation of the ACC has also shown promising results in patients with intractable neuropathic pain [18]. Reduced grey matter volume in pain-regulating brain regions such as the ACC has been attributed to structural ‘maladaptive plasticity’ following chronic nociceptive input [1, 2]. The rostral ACC forms part of the descending inhibitory pathway of the brain’s pain modulation network—a key component of placebo analgesia controlled by the endogenous opioid system [19]. Dysregulation of this system has been suggested as a factor in the presence of pain in fibromyalgic patients [20]. The anti-nociceptive effects of pregabalin have also been shown to involve opioidergic pathways in animal models [21]. Our observation of reduced ACC grey matter volume in people with painful hand OA could, therefore, reflect dysregulation of the descending inhibitory pathway, which the actions of pregabalin and/or duloxetine may help to resolve.

There are limitations inherent to the methodologies used in brain imaging as in our study, including image misregistration due to group differences in brain anatomy and motion artefact, and limitations arising from the small, heterogeneous patient groups included in many studies [14]. Our choice of a region-of-interest analytical approach centred on a strong a priori hypothesis, based on the findings of previous functional MRI studies, as well as morphometric studies in hip OA and other chronic pain conditions [1, 2, 4–7]. Although less biased, a whole-brain approach would have significantly less power due to the small number of subjects in our exploratory study and the large number of brain regions that would be compared. Confirmation of our findings in larger cohorts of patients could pave the way for the development of novel radiological biomarkers of central sensitisation, providing clinicians with objective measures of which patients are likely to benefit from centrally acting analgesic agents.

In conclusion, our study found evidence of reduced ACC grey matter volume in participants with hand OA that persisted after treatment with centrally acting analgesics pregabalin and duloxetine, respectively, despite a significant improvement in NRS clinical pain scores in the pregabalin and duloxetine groups. The ACC is a key pain-processing region of the brain and volume reductions in this brain region in hand OA participants may represent chronic pain-induced neural plasticity. Future longitudinal studies with longer follow-up periods are needed to investigate brain structural changes in chronic painful conditions such as hand OA.

## Electronic supplementary material

Below is the link to the electronic supplementary material.

Supplementary material 1 (DOCX 61 KB)

Supplementary material 2 (DOCX 12 KB)
