# Supplementary material for: Reduced anterior cingulate grey matter volume in painful hand osteoarthritis
Source: Rheumatol Int. 2018 Jun 23;38(8):1429–35. doi: 10.1007/s00296-018-4085-2 (PMC6060828; doi:10.1007/s00296-018-4085-2)
Supplement: Supplementary file 1 — Supplementary material 1 (DOCX 61 KB) [file 296_2018_4085_MOESM1_ESM.docx]

Supplementary Figures

Fig. 1 Boxplot showing the change in NRS pain score between pre-treatment (baseline) and post-treatment (week 13) for each treatment group. Median volumes are displayed in the boxplot, along with upper and lower quartiles. Inner fences represent 1.5 times the interquartile range. ANOVA using a general linear model with repeated measures across all treatment groups gave p = 0.068 for the treatment response. Within each treatment group a paired-t analysis gave p=0.005 for pregabalin (PGN), p =0.05 for duloxetine (DUL) and p=0.151 for placebo (PLA).

Fig. 2 Boxplot showing the change in AUSCAN pain score between pre-treatment (baseline) and post-treatment (week 13) for each treatment group. Median volumes are displayed in the boxplot, along with upper and lower quartiles. Inner fences represent 1.5 times the interquartile range. ANOVA using a general linear model with repeated measures across all treatment groups gave p = 0.021 for the treatment response. Within each treatment group a paired-t analysis gave a significant effect at p=0.01 for pregabalin (PGN), but no significant effects for duloxetine (DUL) and placebo (PLA).
