# Supplementary material for: Reduced anterior cingulate grey matter volume in painful hand osteoarthritis
Source: Rheumatol Int. 2018 Jun 23;38(8):1429–35. doi: 10.1007/s00296-018-4085-2 (PMC6060828; doi:10.1007/s00296-018-4085-2)
Supplement: Supplementary file 2 — Supplementary material 2 (DOCX 12 KB) [file 296_2018_4085_MOESM2_ESM.docx]

**Supplementary Table** Anxiety and depression scores in hand OA participants before and after treatment

| Measure | Duloxetine  (n=11) | Pregabalin  (n=6) | Placebo  (n=11) |
| --- | --- | --- | --- |
| HADS anxiety at baseline, mean (SD) | 6.0 (4.1) | 7.3 (4.5) | 6.5 (3.1) |
| HADS anxiety at 13 weeks, mean (SD); *p*-value for change, relative to baseline | 5.6 (4.0) *p*=0.73 | 5.8 (3.3)  *p*=0.15 | 7.5 (3.9) *p*=0.031 |
| HADS depression at baseline, mean (SD) | 4.4 (3.4) | 7.2 (3.5) | 4.8 (2.4) |
| HADS depression at 13 weeks, mean (SD);  *p*-value for change, relative to baseline | 4.7 (3.7) *p*=0.65 | 5.8 (1.9) *p*=0.29 | 4.7 (2.7) *p*=0.89 |

*HADS*, Hospital Anxiety and Depression Scale. Maximum obtainable score is 21 in the individual anxiety and depression subscales, with mild anxiety/depression = 8-10, moderate anxiety/depression = 11-14, and severe anxiety/depression = 15-21.

**Supplementary Table** NRS and AUSCAN pain scores in hand OA participants before and after treatment

| Measure | Duloxetine  (n=11) | Pregabalin  (n=6) | Placebo  (n=11) |
| --- | --- | --- | --- |
| NRS pain at baseline, mean (SD) | 6.6 (1.6) | 5.8 (1.2) | 6.5 (1.3) |
| NRS pain at 13 weeks, mean (SD); *p*-value for change, relative to baseline | 5.2 (2.7) *p*=0.05 | 3.0 (1.3)  *p*=0.005 | 5.8 (1.9) *p*=0.17 |
| AUSCAN pain at baseline, mean (SD) | 291 (115) | 311 (97) | 315 (51) |
| AUSCAN pain at 13 weeks, mean (SD);  *p*-value for change, relative to baseline | 296 (131) *p*=0.87 | 178 (66) *p*=0.01 | 283 (108) *p*=0.31 |
